# Does DPP-4 inhibitor treatment affect the clinical outcomes of COVID-19 in type 2 diabetes mellitus patients?

**DOI:** 10.14744/nci.2022.34341

**Published:** 2022-07-05

**Authors:** Rumeysa Selvinaz Erol, Esra Cil Sen, Feyza Yener Ozturk, Zeynep Sarac, Gizem Leyla Kokoglu, Muhammed Masum Canat, Duygu Yildiz, Yunus Emre Aytekin, Dilek Yildiz Sevgi, Yuksel Altuntas

**Affiliations:** 1 Department of Endocrinology and Metabolism, University of Health Sciences, Istanbul Sisli Hamidiye Etfal Training and Research Hospital, Istanbul, Turkiye; 2 Department of Internal Medicine, University of Health Sciences Turkey, Sisli Hamidiye Etfal Training and Research Hospital, Istanbul, Turkiye; 3 Department of Infectious Diseases and Clinical Microbiology, University of Health Sciences, Sisli Hamidiye Etfal Training and Research Hospital, Istanbul, Turkiye

**Keywords:** COVID-19, diabetes mellitus, dipeptidyl peptidase-4 inhibitors

## Abstract

**OBJECTIVE:**

We aim to investigate whether the use of dipeptidyl peptidase inhibitors (DPP-4i) affects the severity of disease, hospital mortality, and 3-month post-discharge mortality in type 2 diabetes mellitus (T2DM) individuals with coronavirus disease 2019 (COVID-19) infection.

**METHODS:**

The study included 217 patients with type 2 diabetes hospitalized due to COVID-19 between March and October 2020. The patients included in the study were divided into two groups those using DPP-4i and those not using DPP-4i. Demographic characteristics, laboratory parameters, accompanying risk factors, concomitant comorbidities, hospital mortality, clinical course, and 3-month post-discharge mortality were compared between the patients who used DPP-4i and those who did not use.

**RESULTS:**

The duration of hospitalization was 10.96±9.16 days in the group using DPP-4i, 12.22±9.1 days in the group not using DPP-4i, and when both groups were evaluated together, it was determined as 11.91±9.11 days. The hospitalization periods were similar between DPP-4i users and non-DPP-4i users (p=0.384). The need for mechanical ventilation (p=0.478 OR 0.710 95% confidence interval [CI], 0.274–1.836) and high-flow nasal cannula (p=0.457, OR: 0.331, 95% CI: 0.41–2.67) were similar between DPP-4i users and non-users. It was determined that the mortality (p=0.208, OR: 0.409, 95% CI: 0.117–1.429) and 3-month post-discharge mortality (p=0.383) were similar in the group using DPP-4i and those not using DPP-4i.

**CONCLUSION:**

This study demonstrated that the use of DPP-4i by patients with T2DM in catching COVID-19 does not affect the mortality due to COVID-19, the severity of COVID-19 disease, and 3-month post-discharge mortality.

## Highlight key points

- The use of DPP-4i by patients with T2DM before COVID-19 does not affect the mortality due to COVID-19.
- The use of DPP-4i by patients with T2DM before COVID-19 does not affect the severity of COVID-19 disease.
- The use of DPP-4i by patients with T2DM before COVID-19 infection does not affect 3-month post-discharge mortality.
- The mortality due to COVID-19 in T2DM is 11%.

Coronavirus disease 2019 (COVID-19) pandemic is an unextinguished fire that has spread worldwide since reported in China in December 2019, infected more than 243 million people, and caused radical change and transformation in the world order [1]. Diabetes mellitus type 2 is a well-known risk factor for Covid-19 mortality, which can lead to more severe illness and acute respiratory distress syndrome (ARDS). It has been believed that elevated DPP-4 expression in type 2 diabetes mellitus is the cause of high disease morbidity and mortality in Covid-19. Also, DPP-4 is defined as one of the spice glycoprotein receptors for severe acute respiratory syndrome coronavirus-2 (SARS-CoV2). Due to these, DPP-4 inhibitors (DPP-4i) have sparked an interest [2–6].

DPP-4 was first shown in 2013 to be a functional receptor that provides entry to cells through the S1b domain of spike glycoprotein of Middle East respiratory syndrome coronavirus (MERS-CoV), which is genetically similar to COVID-19 [5, 7]. It is known that angiotensin-converting enzyme (ACE)2 is the SARS-CoV-2 spike glycoprotein receptor [8]. A study demonstrated a correlation between DPP-4 and ACE2, suggesting that both membrane proteins are involved in the pathogenesis of virus entry [9]. Coexpression of ACE2 and DPP4/CD26 as the receptors of spike glycoproteins may lead to hypothesizing that different human coronaviruses (CoV) target similar cells types in various human tissues and may explain the presence of similar clinical features in infected patients. It is believed that DPP-4 also functions as a receptor for the viral entry of SARS-CoV-2, which is genetically identical to MERS-CoV [4, 5, 10]. DPP-4 T-cell antigen, also known as CD26, is a cell surface protein expressed in many tissues and a soluble molecule found in serum/plasma fluids, as well [11, 12]. DPP-4 also plays a role in the immune system, besides providing glucose regulation by causing the degradation of incretins and increasing insulin secretion.

Furthermore, DPP-4i has been shown to have significant systemic anti-inflammatory effects, reducing the concentration of major inflammatory cytokines such as interleukin-6 (IL-6) [13]. Furthermore, in mouse models, sitagliptin, which is DPP-4i, has been found to suppress the lung injury caused by lipopolysaccharide by reducing the release of cytokines such as tumor necrosis factor-alpha and IL-6, and significantly reducing the hyper-pulmonary inflammation in acute lung injury [14]. DPP-4i promotes faster tissue healing by increasing stromal cell-derived factor (SDF)-1 levels [15, 16].

Accumulating evidence suggests that increased pro-inflammatory cytokine release, known as the “cytokine storm” triggered by the host immune response to SARS-CoV-2, is directly associated with the poor prognosis of COVID-19 [17, 18]. In this context, the hypothesis of whether DPP-4 inhibition increases susceptibility to COVID-19 infection and whether it reduces significant complications such as ARDS, which represents the leading cause of death in COVID-19 patients, has been brought up [19].

Among diabetic patients with COVID-19, the outcomes regarding the risk of progression to more severe forms of the disease and mortality rates between DPP-4i users and non-users are heterogeneous. While some of the studies have concluded that DPP-4i has positive effects on COVID-19 mortality and severe illness due to COVID-19 [20–22], some others have revealed that the use of DPP-4i has neutral or harmful effects[23–29] on these two phenomena. Our study aimed to investigate whether the use of DPP-4i affects severe disease, hospital mortality, and 3-month post-discharge mortality in T2DM individuals with COVID-19 infection.

## MATERIALS AND METHODS

It is a single-center, case-controlled, retrospective observational study. It was carried out with the approval of the ethics committee (no; 1760, date; December 22, 2020) of Sisli Hamidiye Etfal Training and Research Hospital by the ethical rules of the Declaration Helsinki and the national regulations on retrospective observational studies. Due to the retrospective study design, the requirement for informed consent was waived by the hospital ethics committee. Our study was also reviewed and approved by the Ministry of Health.

The data of 2196 patients hospitalized in Sisli Hamidiye Etfal Training and Research Hospital between March 2020and October 2020 due to COVID-19 infection were analyzed. In this analysis, patients with T2DM were chosen based on their medical history or use of antihyperglycemic agents or DM criteria in the ADA guidelines [30].

Two hundred and seventeen patients with T2DM who were confirmed to have COVID-19 according to the reverse transcription-polymerase chain reaction test in nasal or pharyngeal swab samples were included in the study. Those with T1DM diagnosis and those who were younger than 18 years of age were excluded from the study. No other patient selection was applied in our research, and the patients of all ages and genders over 18 years of age were included in the study.

Data were obtained from electronic medical records. Demographic characteristics of patients such as age, gender, and body mass index (BMI), accompanying risk factors such as smoking, hypertension (HT), hyperlipidemia (HL), comorbidities such as cerebrovascular disease (CVD), coronary artery disease (CAD), chronic obstructive pulmonary disease (COPD), and cancer were recorded. In addition, laboratory parameters such as glucose, HbA1c creatinine (Cr), alanine aminotransferase, aspartate aminotransferase, lactate dehydrogenase (LDH), C-reactive protein (CRP), ferritin, neutrophil/lymphocyte, lymphocyte, d-dimer, and procalcitonin (PCT) at the time of admission to the hospital were also recorded. Furthermore; data regarding the use of DPP-4i, oral antidiabetic drugs such as sulfonylurea, basal and bolus insulin, antihypertensive drugs such angiotensin receptor blockers, ACE inhibitor, Ca channel blocker, beta-blocker, and data on the presence of an antiplatelet or warfarin use were collected before hospitalization.

Participants were divided into two groups regarding DPP-4i users and non-DPP-4i users. DPP-4i users were defined as patients using DPP-4i for at least 3 months before hospital admission to avoid including patients who were started on DPP-4i at discharge.

The total length of hospital stay was determined. Discharged patients were called one by one, and patients who died within 3 months after discharge were determined. Patients who needed mechanical ventilation and a high-flow nasal cannula were evaluated as severe illness due to COVID-19. The primary endpoint of the study was the need for mechanical ventilation and high-flow nasal cannula, the secondary endpoint was in-hospital mortality, and the tertiary endpoint was 3 months post-discharge mortality.

### Statistical Analysis

Statistical analyses were performed in the IBM SPSS Version 25 program (IBM SPSS, Armonk, NY). Continuous data were shown as a mean and standard deviation; categorical data were established as percentage and frequency ratios. Suitable parametric or non-parametric tests were chosen according to whether the data showed normal distribution or not. In the comparison of independent groups, the Chi-square test was used for categorical ones, and independent sample t-test and Mann–Whitney U-test were used for continuous data. Multinomial logistic regression analysis was used to evaluate some parameters whose effects on severe disease and mortality were investigated. Kaplan–Meier and Cox regression analyses were used to compare treatments for severe disease states and mortality. To improve the data, considering the probabilities in binary logistic regression analysis regarding age, BMI, and gender data, DPP-4i using and non-using individuals were matched with the equivalent or the closest scores through the obtained propensity score matching (PSM), and paired analyses were performed. Continuous data of dependent groups were evaluated using paired sample t-test and Wilcoxon signed-ranks test. In the PSM group, parameters were evaluated with regression, Cox regression, and Chi-square tests, as were done with the unmatched data. P<0.05 was considered as statistically significant.

## RESULTS

A total of 217 patients with T2DM, diagnosed with COVID-19 infection, were included in the study. Fifty-three of these were using DPP-4i. The primary demographic and clinical characteristics of both patient groups are shown in Table 1. The mean age of patients using DPP-4i was 63.9±12.2, while the mean age of patients not using DPP-4i was 62.0±12.9. There was no statistical difference between the two groups regarding age, gender, and BMI (p=0.339, p=0.325, and p=0.847, respectively). No difference was observed between the two groups in terms of other demographic characteristics (Table 1). We observed that the participants were relatively homogeneous when their demographic characteristics and vital findings were considered.

**Table 1:** Demographic and clinical characteristics of patients

	On DPP-4i Mean±SD (n=53)	Not on DPP-4i Mean±SD (n=164)	Total patients Mean±SD (n=217)	p
Age (years)	63.9±12.2	62.0±12.9	62.5±12.7	0.339
Female/male	24/29	87/77	111/106	0.325
BMI (kg/m^2^)	30.49±6.52	29.53±5.50	29.99±5.76	0.847
SBP (mmHg)	125.81±16.82	124.18±16.27	124.58±16.38	0.34
DBP (mmHg)	76.7±9.06	75.07±10.01	75.49±9.8	0.357
HR	84±14	88.26±12.77	85.9±13.07	0.339
RR	17.4±3.46	18.22±3.73	18.01±3.67	0.091
O_2_ saturation	94.19±3.94	94.05±4.3	94.09±4.21	0.85
Smoker	13 (24.5%)	33 (20.12%)	46 (21.19%)	0.477
Comorbidities (%)				
Hypertension	60.4	64.6	63.6	0.576
CAD	20.8	27.4	25.8	0.334
CVD	7.5	3	4.1	0.227
COPD	7.5	6.7	6.9	0.834
Hyperlipidemia	41.5	28.7	31.8	0.081
Cancer	1.9	5.5	4.6	0.457
Medication (%)				
Basal insulin	37.7	37.2	37.3	0.944
Bolus insulin	24.5	31.1	29.5	0.362
Sulfonylurea	15.1	11	12	0.422
Metformin	81.1	61.6	66.4	**0.009**
Thiazolidinedione	5.7	4.9	5.1	0.732
SGLT-2 inhibitor	20.8	4.3	8.3	**0.00**
ACE	20.8	22.8	22.3	0.752
ARB	17	14.6	15.2	0.679
Ca channel blocker	15.1	27.4	24.4	0.069
Beta-blocker	32.1	27	28.2	0.475
Antiplatelet	34	31.7	32.3	0.760
Warfarin	0.0	3.7	2.8	0.340
Laboratory findings				
Glucose (mg/dl)	199.23±75.88	192.77±91.89	194.32±88.17	0.278
Hba1c (%)	8.71±2.22	8.29±2.13	8.37±2.14	0.423
Lymphocytes (10^3^/μ/L)	1390.57±693.17	1649.70±3401.06	1586.41±2976.09	0.864
Neutrophil/lymphocytes	4.19±3.57	4.83±4.67	4.67±4.43	0.752
ALT	30.51±28.01	30.60±32.29	30.57±31.23	0.962
AST	34.62±23.73	34.33±23.22	34.40±23.29	0.918
LDH	274.00±104.15	296.62±114.65	290.88±112.26	0.163
Cr (mg/dl)	0.98±0.41	1.11±1.06	1.08±0.94	0.410
CRP (mg/dl)	65.70±60.28	70.35±68.02	69.22±66.11	0.897
Ferritin	317.13±436.31	356.40±572.54	346.44±540.52	0.831
D-dimer	884.16±870.31	1082.01±1318.14	1034.25±1226.06	0.307
Procalcitonin (ng/ml)	0.28±0.70	1.13±8.30	0.92±7.26	0.635

SD: Standard deviation; BMI: Body mass index; SBP: Systolic blood pressure; DBP: Diastolic blood pressure; HR: Heart rate; RR: Respiratory rate; CAD: Coronary artery disease; CVH: Cerebrovascular disease; COPD: Chronic obstructive pulmonary disease; SGLT: Sodium-glucose cotransporter; ACE: Angiotensin-converting enzyme; ARB: Angiotensin receptor blocker; ALT: Alanine aminotransferase; AST: Aspartate aminotransferase; LDH: Lactate dehydrogenase; Cr: Creatinine; CRP: C-reactive protein; DPP-4i: Dipeptidyl peptidase inhibitor.

There was no difference between the two groups in terms of systemic blood pressure, vital findings such as HR max, and laboratory tests (Table 1). There was no difference between the two groups in terms of other comorbidities such as COPD, CVD, CAD, HT, HL, and cancer. As expected, treatment of patients using DPP-4i was often accompanied by metformin and/or sodium-glucose cotransporter-2 inhibitors (SGLT-2i) in accordance with the Society of Endocrinology and Metabolism Society guidelines [31] (p=0.00 and p=0.009, respectively). While SGLT-2i use was 20.8% (n=11) and metformin was 81.1% (n=43) in DPP-4i users, the ratios were 4.3% (n=7) for SGLT-2i and 61.6% (n=101) for metformin in non-DPP-4i users (Table 1).

About 9.8% (n=217/2196) of the patients hospitalized due to COVID-19 infection had T2DM, and the mortality of these T2DM patients was 11.1% (n=24). The duration of hospitalization was 10.96±9.16 days in the group using DPP-4i, 12.22±9.1 days in the group not using DPP-4i, and when both groups were evaluated together, it was determined as 11.91±9.11 days. The hospitalization period was similar between DPP-4i users and non-DPP-4i users (p=0.384).

The need for mechanical ventilation (p=0.478 odds ratio [OR] 0.710 95% confidence interval [CI], 0.274–1.836), and high-flow nasal cannula (p=0.457, OR: 0.331, 95% CI: 0.41–2.67) were similar between DPP-4i users and non-users. It was determined that the mortality was similar in the group using DPP-4i and those not using (p=0.208, OR: 0.409, 95% CI: 0.117–1.429) (Table 2, 3).

**Table 2:** Duration and outcomes with T2DM and COVID-19 treated with DPP-4i and without DPP-4i

	On DPP-4i	Not on DPP-4i	Total patients	p
Duration				
The time between first symptom and hospitalization, median (days)	4.19±2.84	4.74±3.87	4.61±3.64	0.483
Hospitalization time, median (days)	10.96±9.16	12.22±9.10	11.91±9.11	0.384
Outcome (%)				
High-flow nasal cannula	1.9	5.5	4.6	0.457
Mechanic ventilation	11.3	15.2	14.3	0.478
Death	5.7	12.8	11.1	0.208
3-month post-discharge mortality	6	3.49	4.14	0.383

Total means, using DPP-4 inh and not using DPP-4 inh. T2DM: Type 2 diabetes mellitus; DPP-4i: Dipeptidyl peptidase inhibitor.

**Table 3:** Outcomes in patients with T2DM and COVID-19 treated with DPP-4i and without DPP-4i

	Propensity score match		Unmatched uncorrected	
	OR+95% CI	p	OR+95% CI	p
High-flow nasal cannula	0.236 (0.02–2.18)	0.363	0.331 (0.41–2.67)	0.457
Mechanic ventilation	1 (0.30–3.32)	1	0.710 (0.27–1.83)	0.478
Death	0.576 (0.13–2.54)	0.716	0.409 (0.11–1.42)	0.208
3-month post-discharge mortality	0.845 (0.58–1.20)	0.811	0.614 (0.22–2.15)	0.383

T2DM: Type 2 diabetes mellitus; DPP-4i: Dipeptidyl peptidase inhibitor; OR: Odds ratio; CI: Confidence interval.

OR values for the need for high-flow nasal cannula, and mechanical ventilation, mortality in hospitalization, and 3 months post-discharge mortality were similar before and after PSM (Table 3).

No correlation was found for CRP, LDH, lymphocyte, D-dimer, ferritin, and PCT values measured at the time of admission to the hospital, with in-hospital mortality and for the need for high-flow nasal cannula and mechanical ventilation.

## DISCUSSION

We found that the mortality rate, the need for a high-flow nasal cannula, and the need for mechanical ventilation were similar between the patients who used DPP-4i and did not use DPP-4i. According to the results of our study, using DPP-4i before COVID-19 infection does not affect the mortality due to COVID-19 and the severity of COVID-19 infection. About 9.8% of the patients hospitalized due to COVID-19 infection had T2DM, and the mortality rate of these T2DM patients was 11.0%.

In a population-based study conducted in the UK that included 2.85 million T2DM patients, it was found that the use of DPP-4i slightly increased the risk of mortality [29]. They attributed this increased risk to prescribing DPP-4i mostly in older and more fragile patients. Advanced age is an independent risk factor for clinical outcomes associated with COVID-19. When we look at the study, we see that more than 50% of the patients using DPP-4i are reported to be over 65 years old. In our study, the mean age of the patients who used and did not use DPP-4i was below 65 years.

Contrary to our study, in a population-based study carried out in Turkey, including 9100 patients, it was observed that the use of DPP-4i (OR 0.57) decreased the mortality rate due to COVID-19 when compared to those who did not use DPP-4i [21]. In the study, the Cr clearance of those who did not use DPP-4i was found to be statistically lower than those who used DPP-4i. In our study, there was no statistical difference between the Cr values of the patients. Lower Cr clearance in non-DPP-4i users may be the reason for the increased mortality in non-DPP-4i users compared to DPP-4i users. In addition, the small number of patients in our study, different demographic characteristics, and concomitant comorbidities may have caused our results to be different.

Regarding T2DM patients with COVID-19 infection, the Sitagliptin treatment group and the group receiving standard treatment were compared in a case-controlled non-randomized study called sitagliptin in diabetic patients with COVID-19, which differs from our study in that the use of DPP-4i was used during the hospitalization period, not before hospitalization. It was shown that mortality in the group using sitagliptin, which is a DPP-4i (hazard ratio 0.44), decreased when compared to the standard treatment group [22]. DPP-4i therapy during Covid-19 infection may have improved the study results (Covid-19 related mortality and morbidity). Consistent with our study, in an observational study conducted in France, including 1166 patients, it was determined that there was no relationship between the use of DPP-4i and mortality due to COVID-19 [28]. The lack of positive effects on mortality due to COVID-19 and severe disease related to COVID-19 may be due to the ineffectiveness of DPP-4i on the soluble form of DPP-4.

Due to the heterogeneity of studies evaluating the effects of DPP-4i use on death and severe illness due to COVID-19, various meta-analyses have been made in a short time. Two meta-analyses revealed that there was no significant difference occurring with the use of DPP-4 inhibitors before hospital admission regarding the risk of developing a severe or fatal disease course in COVID-19 patients (risk ratio: 1.15; 95% CI: 0.64–2.06, risk ratio: 0.81; 95% CI: 0.57–1.15) [32, 33]. In the recent meta-analysis, which examined the most significant number of studies to date, 66,914 patients were evaluated and it was determined that the use of DPP-4i had no statistical significance on mortality [34].

The effects of DPP-4i on in-hospital morbidity and mortality due to COVID-19 have been the main focus. There are no studies on the long-term consequences of the use of DPP-4i on the health of discharged patients. Multiple organ damage affecting the liver, kidneys, and heart, which can eventually result in organ failure, is quite common among COVID-19 patients in intensive care units [35]. SARS-CoV-2 either induces new cardiac pathologies such as myocarditis and/or exacerbates the existing ones [36], and this points to the potential long-term cardiovascular effects of COVID-19. These phenomena may be important for diabetic patients who already have reduced lung, heart, and kidney function and may make them particularly susceptible to cumulative organ damage during SARS-CoV-2 infection [37]. Recently, Smelcerovic et al. [37] suggested that DPP-4i may be effective in the prevention and treatment of pulmonary fibrosis, heart, and kidney damage. They claimed that DPP-4i will achieve these effects by increasing SDF-1, which provides an increase in endothelial progenitor cells, improves the result of functional myocardial reparation, and has pleiotropic anti-inflammatory effects. For these reasons, although it was not objective, the wellness of the patients in the 3-month post-discharge period was questioned in our study. The 3-month post-discharge mortality of those who used DPP-4i and those who did not use DPP-4i were found to be similar.

Depending on the cytokine storm caused by the excessive immune response, inflammatory markers such as PCT, ferritin, D-dimer, and CRP may increase in patients with COVID-19, depending on the severity of the disease [38–40]. We did not detect any difference in inflammatory markers such as PCT, ferritin, and CRP between the patients who used DPP-4i and those who did not use it.

Our study has several limitations. First and foremost is that the small number of patients may have prevented our statistical significance. In addition, being a single-centered study with an observational design is the other limitation of our study. However, when the data in our study are evaluated, the patients using and not using DPP-4i are relatively homogeneous in terms of demographic characteristics, concomitant comorbidity, laboratory findings, and diabetes regulation. The homogeneity of the groups is a strong feature of our study.

## Conclusion

As a result, this study demonstrated that the use of DPP-4i by patients with T2DM in catching COVID-19 does not affect the mortality due to COVID-19, the severity of COVID-19 disease, and 3-month post-discharge mortality.
